# Reduced Serum sRAGE Levels Identify COPD and Reflect Disease Severity: Findings from a Cross-Sectional Study in India

**DOI:** 10.3390/diagnostics15222910

**Published:** 2025-11-17

**Authors:** Venkateshkumar Chandanna Seri, Mohammed Kaleem Ullah, Jayaraj Biligere Siddaiah, Sindaghatta Krishnarao Chaya, Komarla Sundararaja Lokesh, Suhail Azam Khan, Aishwarya R. Aladakatti, Shamnaz Shahul, Vivek Vasanthan, Medha Karnik, SubbaRao V. Madhunapantula, Swaroop Ramaiah, Sachith Srinivas, Vikhnesh Padmakaran, Malavika Shankar, Ashwaghosha Parthasarathi, Padukudru Anand Mahesh

**Affiliations:** 1 Department of Respiratory Medicine, JSS Medical College, JSS Academy of Higher Education and Research, Mysuru 570015, India; venkateshseri212@gmail.com (V.C.S.); drjayarajbs@yahoo.com (J.B.S.); chaya.sindaghatta@gmail.com (S.K.C.); lokeshpulmo@gmail.com (K.S.L.); khan2201@gmail.com (S.A.K.); aishwaryaaladakatti@gmail.com (A.R.A.); shamnazshahul994@gmail.com (S.S.); vivekvasanthan0@gmail.com (V.V.); 2 Center of Excellence in Molecular Biology and Regenerative Medicine (CEMR) Laboratory (DST-FIST Supported Center and ICMR Collaborating Center of Excellence—ICMR-CCoE), Department of Biochemistry (DST-FIST Supported Department), JSS Medical College, JSS Academy of Higher Education and Research, Mysuru 570015, India; ka7eem@gmail.com (M.K.U.); medhakarniksr@ymail.com (M.K.); mvsstsubbarao@jssuni.edu.in (S.V.M.); 3 Thomas Memorial Hospital, WVU Medicine, South Charleston, WV 25309, USA; swaroop2408@gmail.com; 4 Respiratory Medicine, Barnsley NHS Foundation Trust, South Yorkshire S75 2EP, UK; sachithgowda043@gmail.com; 5 General Medicine, Barnsley NHS Foundation Trust, South Yorkshire S75 2EP, UK; drvikhneshpadmakaran@gmail.com; 6 Bridgeport Hospital, Yale New Haven Health, Bridgeport, CT 06610, USA; malavika18.shankar@gmail.com; 7 Rutgers RWJ Barnabas Center for Climate, Health and Healthcare, New Brunswick, NJ 08901, USA; ashwa.partha@gmail.com

**Keywords:** COPD, sRAGE, biomarker, tobacco smoke, biomass smoke, disease severity

## Abstract

**Background:** Chronic Obstructive Pulmonary Disease (COPD) remains a leading cause of morbidity and mortality worldwide, particularly in low- and middle-income countries (LMICs), where both tobacco and biomass smoke exposure are major risk factors. While spirometry is the diagnostic gold standard, reliable non-invasive biomarkers are needed for early detection and disease monitoring. The soluble receptor for advanced glycation end-products (sRAGE), a circulating decoy receptor with anti-inflammatory activity, has shown potential in this context. **Methods:** In this prospective, exposure-stratified, cross-sectional study, 150 adults were enrolled into four groups of 25 each—tobacco-smoke COPD, male tobacco-exposed controls, biomass-smoke COPD, and female biomass-exposed controls—along with 50 healthy controls (25 males, 25 females). Participants underwent clinical evaluation, spirometry, and serum sRAGE quantification (ELISA). Systemic inflammation was assessed using neutrophil-to-lymphocyte ratio (NLR) and platelet-to-lymphocyte ratio (PLR). Correlation and receiver operating characteristic (ROC) analyses determined diagnostic performance. **Results:** Serum sRAGE levels were significantly lower in tobacco-induced (545 ng/mL) and biomass-induced COPD (540 ng/mL) versus controls (1207–1462 ng/mL; *p* < 0.001). sRAGE correlated positively with FEV_1_, FVC, and FEV_1_/FVC (r = 0.54–0.75, *p* < 0.001), and negatively with CAT, mMRC, and SGRQ-C. ROC analysis showed excellent discrimination (AUC = 0.990; 94% sensitivity; 96% specificity at 946 ng/mL cutoff). **Conclusions:** Serum sRAGE is a robust, non-invasive biomarker for COPD diagnosis and severity assessment across exposure phenotypes. Its integration into clinical practice may enhance early detection and risk stratification, particularly in LMICs.

## 1. Introduction

Chronic Obstructive Pulmonary Disease (COPD) imposes a growing global health burden, ranking as the third leading cause of death worldwide, with over 3 million annual fatalities [1,2,3]. This progressive and irreversible disease is typified by persistent airflow limitation and chronic lung inflammation in response to noxious exposures such as tobacco and biomass smoke [4,5,6]. Beyond respiratory morbidity, COPD contributes significantly to systemic consequences, including cardiovascular comorbidity, cachexia, and metabolic derangements [7,8].

In high-income countries, tobacco smoking remains the major risk factor for COPD. However, in low- and middle-income countries (LMICs)—particularly in rural settings—household biomass smoke from cooking and heating is a pervasive, yet under-recognized, driver of disease [3,6,9]. Biomass-COPD disproportionately affects women and manifests a distinct airway-predominant phenotype, often with less emphysema but more small-airway fibrosis and bronchial wall thickening compared to tobacco-related disease [1,3,10]. This divergence suggests differing pathogenic mechanisms and reinforces the need for phenotype-specific diagnostic strategies [8,10,11].

Spirometry, the primary diagnostic test for COPD (defined by post-bronchodilator FEV_1_/FVC < 0.70), is infrequently deployed in primary care, especially in LMICs, and lacks sensitivity for early or non-emphysematous phenotypes [3,6]. Moreover, current clinical tools (e.g., symptom scores) inadequately capture underlying pathophysiology and predict progression.

Consequently, significant effort has been directed towards blood-based biomarkers. Among these, soluble receptor for advanced glycation end-products (sRAGE) has emerged as a robust candidate. Acting as a decoy receptor that neutralizes pro-inflammatory ligands, sRAGE levels are consistently decreased in COPD, particularly in emphysema-predominant phenotypes [12]. Major cohort studies—including COPDGene, SPIROMICS, and ECLIPSE—demonstrate that lower sRAGE correlates with reduced airflow, greater emphysema on imaging, and faster lung function decline [8]. Integrative genomics studies further suggest that sRAGE may be causally protective and modifiable through RAGE antagonism [13].

Despite its promise, existing sRAGE research has largely focused on tobacco-related COPD in Western populations, with limited exploration of biomass-related disease, which affects over 2.6 billion people globally [9,14]. The pathophysiology of biomass-related COPD, including its inflammatory milieu and structural patterns, differs from tobacco-induced disease [10,11]. Notably, the comparative performance of sRAGE in biomass-COPD vs. tobacco-COPD has not been established.

India, with its extensive dual exposures and high COPD burden, offers a unique setting to investigate sRAGE. This study evaluates serum sRAGE across five groups—tobacco-COPD, biomass-COPD, respective exposure-matched controls, and healthy individuals—examining its associations with lung function, symptom severity, systemic inflammation, and diagnostic accuracy. Our hypothesis posits that sRAGE will be significantly reduced in COPD, irrespective of exposure type, and may discriminate disease presence and severity across phenotypes, supporting its role in early detection and phenotype-tailored care in resource-limited settings.

## 2. Materials and Methods

### 2.1. Study Design and Setting

This was a prospective, exposure-stratified, cross-sectional study conducted over 18 months (June 2023 to December 2024) at the Department of Respiratory Medicine, JSS Hospital, Mysuru—a tertiary care teaching hospital in southern India. The study was designed to compare clinical, spirometric, inflammatory, and biomarker profiles among COPD patients with differing exposure histories, along with appropriate control groups. All participants were recruited and evaluated using standardized clinical protocols within a single center, allowing consistent data collection across groups.

### 2.2. Participants

A total of 150 adults were enrolled and evenly distributed across five predefined groups (*n* = 25 per group): (1) male smokers with COPD (TS-COPD), (2) female biomass-exposed individuals with COPD (BMS-COPD), (3) male smokers without COPD (TS-Control), (4) female biomass-exposed individuals without COPD (BMS-Control), (5) healthy non-smoking controls (25 males, 25 females).

Participants with COPD were aged ≥40 years, had relevant exposure histories (tobacco or biomass smoke), and met the Global Initiative for Chronic Obstructive Lung Disease (GOLD) diagnostic criteria, including a post-bronchodilator FEV_1_/FVC ratio < 0.70. Exposure-matched control groups (TS-Control and BMS-Control) consisted of individuals with similar exposure profiles but without airflow obstruction or respiratory symptoms. The healthy control group comprised individuals without any history of tobacco or biomass smoke exposure and with normal spirometry. All participants provided written informed consent.

Exclusion criteria included the presence of other chronic respiratory diseases (such as asthma, bronchiectasis, or interstitial lung disease), recent or ongoing infections, malignancy, significant systemic illness (such as *Diabetes mellitus*, hypertension, renal disease ischemic heart disease, and liver disease), ongoing corticosteroid therapy, and refusal to provide informed consent.

### 2.3. Clinical and Functional Assessments

Symptom burden was quantified using validated, interviewer-administered tools: the COPD Assessment Test (CAT), the Modified Medical Research Council (mMRC) Dyspnea Scale, and the St. George’s Respiratory Questionnaire for COPD (SGRQ-C). These instruments provided robust measures of symptom intensity, functional limitation, and health-related quality of life.

Pulmonary function testing (PFT) was performed using the NDD EasyOneSpirometer (ndd Medical Technologies, Zurich, Switzerland) in accordance with American Thoracic Society/European Respiratory Society (ATS/ERS) standards. The parameters recorded included forced expiratory volume in one second (FEV_1_), forced vital capacity (FVC), and the FEV_1_/FVC ratio. Assessments were conducted both before and after administration of a bronchodilator to confirm airflow limitation and to classify severity according to the Global Initiative for Chronic Obstructive Lung Disease (GOLD) criteria.

### 2.4. Exposure Assessment

Detailed exposure histories were obtained. Smoking status was documented in pack-years and smoking index, including age of initiation and, if applicable, cessation. For biomass-exposed participants, daily exposure duration, cumulative years of exposure, and a composite biomass exposure index were computed to quantify intensity and chronicity of inhalant risk [15].

### 2.5. Laboratory and Biomarker Evaluations

Systemic inflammation was characterized via neutrophil-to-lymphocyte ratio (NLR) and platelet-to-lymphocyte ratio (PLR), calculated from complete blood counts using 5-part differential hematology analyzers (Mindray BC series (Mindray Bio-Medical Electronics Co., Ltd., Shenzhen, China) and Sysmex XN 1000 automated blood analyzer (Sysmex Corp., Kobe, Japan)). A total of 5ml of venous blood was obtained from each subject under aseptic precautions, allowed to clot for 30 min, and then centrifuged at room temperature. The isolated serum was stored in Eppendorf tubes and kept at −80 °C until the sRAGE assays were performed. Serum sRAGE levels were assessed using a sandwich ELISA kit (FineTest^®^, Cat. No. EH0408, Wuhan Fine Biotech Co., Ltd., Wuhan, China). Each specimen was tested in duplicate after establishing a calibration curve and applying rigorous quality controls as per manufacturer specifications.

### 2.6. Data Collection Procedure

Data collection was performed in a single, structured hospital visit to minimize attrition risk. Trained personnel conducted the interviews and administered questionnaires in accordance with standardized protocols. Spirometry was conducted with appropriate clinical monitoring. Venous blood samples were processed promptly to uphold sample integrity.

### 2.7. Sample Size

The sample size for this study was determined based on the primary objective of assessing differences in serum sRAGE levels between COPD patients and control groups across distinct exposure phenotypes (tobacco smoke and biomass smoke). Previous studies assessing sRAGE in COPD reported large effect sizes with mean differences exceeding one standard deviation between patients and healthy controls. Assuming a moderate to large effect size (Cohen’s d = 0.8), a significance level (alpha) of 0.05, and a desired statistical power of 0.80 for two-sided hypothesis testing, a minimum of 25 subjects per group was calculated to reliably detect significant differences in sRAGE levels between groups using ANOVA or non-parametric equivalents. The chosen sample size also allows reasonable precision for correlation analyses between sRAGE and lung function or symptom scores, as well as ROC curve evaluation of diagnostic accuracy.

### 2.8. Statistical Methods

All statistical analyses were performed using SPSS (version 25.0; IBM Corp., Armonk, NY, USA) and Jamovi (version 2.6.26, The jamovi Project, SYD, AUS). Continuous variables were assessed for normality using the Shapiro–Wilk test and summarized as means ± standard deviations (for normally distributed data) or medians with interquartile ranges (for non-normally distributed data). Categorical variables were summarized as frequencies and percentages.

Between-group comparisons for continuous variables were conducted using one-way analysis of variance (ANOVA), followed by Scheffé’s post hoc test for pairwise comparisons. Where assumptions for parametric testing were not met, the Kruskal–Wallis and Dunn tests were applied. Associations between sRAGE and clinical parameters—including lung function indices, symptom scores, and inflammatory markers—were evaluated using Pearson or Spearman correlation coefficients, as appropriate.

The diagnostic utility of serum sRAGE in identifying COPD and differentiating between severity grades was assessed using receiver operating characteristic (ROC) curve analysis. The area under the curve (AUC), sensitivity, specificity, positive predictive value (PPV), negative predictive value (NPV), optimal cutoff points (Youden’s Index), and 95% confidence intervals were calculated for each comparison. A two-sided *p*-value < 0.05 was considered statistically significant for all analyses.

### 2.9. Ethical Considerations

The study was approved by the Institutional Ethics Committee of our institution. (IEC approval number JSS/MC/PG/2046/108/2023-24 dated 23 June 2023) in accordance with the Declaration of Helsinki. All participants provided written informed consent. Privacy and confidentiality were ensured throughout the study

## 3. Results

### Baseline Characteristics

The study cohort included five groups: TS-COPD (tobacco-smoking-related COPD), TS-CONTROL (tobacco-smoking controls), BMS-COPD (biomass-smoke-related COPD), BMS-CONTROL (biomass smoke controls), and healthy controls. The average age was highest in the BMS-COPD group (68.00 ± 7.74 years) and lowest among healthy controls (50.52 ± 9.92 years). BMI was comparatively similar across all groups, ranging from 21.60 to 23.62 kg/m^2^. Each COPD group consisted of 25 subjects, with the TS groups being entirely male and the BMS groups entirely female, while healthy controls had equal gender representation. Lung function, measured by FEV_1_/FVC ratio and percent predicted FEV_1_ and FVC, was significantly reduced in both COPD groups compared to their respective controls and healthy individuals. Biomass exposure and BMEI were relevant only for the BMS groups, while smoking-related indices (pack-years and smoking index) were present in the TS groups. COPD severity was categorized using GOLD grading, with patients distributed across mild to severe grades. Symptom scores (CAT, mMRC, and St. George’s questionnaires) were highest in COPD groups, indicating greater disease impact. Inflammatory markers, notably neutrophil-to-lymphocyte ratio (NLR) and platelet-to-lymphocyte ratio (PLR), were elevated, especially in BMS-COPD, indicating systemic inflammation. Soluble RAGE (sRAGE) levels were markedly lower in both COPD groups compared to controls, with the highest levels noted in healthy individuals, implying a potential role in disease pathogenesis or severity (Table 1). Additional analysis on gender-based stratification of sRAGE levels is provided in Supplementary Table S1.

sRAGE concentration demonstrates strong positive correlations with key lung function parameters, including the FEV_1_/FVC ratio, FVC percent predicted, and FEV_1_ percent predicted, indicating that higher sRAGE levels are associated with better pulmonary function (all *p* < 0.001). In contrast, sRAGE shows strong negative correlations with clinical symptom and impact scores such as the CAT score, mMRC grade, and SGQRC total, suggesting that higher sRAGE levels are linked to fewer symptoms and lower disease impact (all *p* < 0.001). Additionally, sRAGE levels exhibit a weak negative correlation with NLR (*p* < 0.001) and a weak, non-significant negative correlation with PLR (*p* = 0.072), suggesting limited and possibly negligible associations with these inflammatory markers. Overall, higher sRAGE concentrations are closely related to better respiratory health and reduced symptom burden in this population (Table 2).

**Figure 1:**
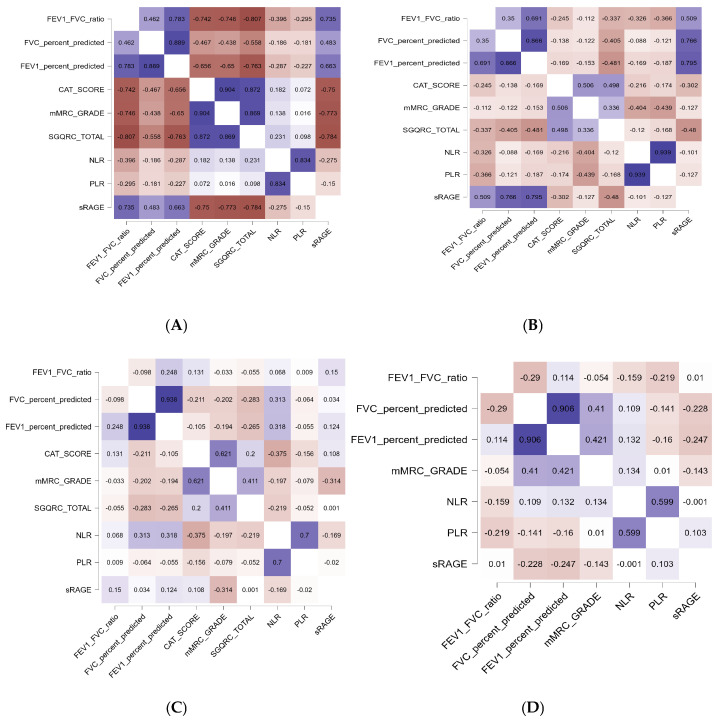
Correlation heatmaps showing associations between serum sRAGE concentrations and demographic, spirometric, symptom, and inflammatory variables across study groups: (**A**) All subjects, (**B**) COPD group, (**C**) TS-control + BMS-control, (**D**) healthy controls. Abbreviations: FEV_1_: Forced Expiratory Volume in One Second; FVC: Forced Vital Capacity; CAT: COPD Assessment Test; mMRC: Modified Medical Research Council; SGQRC: COPD-Specific Version of the St. George’s Respiratory Questionnaire; NLR: Neutrophil to Lymphocyte Ratio; PLR: Platelet to Lymphocyte Ratio; sRAGE: Soluble Receptor for Advanced Glycation End-Products; COPD: Chronic Obstructive Pulmonary Disease; TS: Tobacco Smoke; BMS: Biomass Smoke. Note: Color intensity represents the strength and direction of the correlation, with blue indicating positive correlations and red indicating negative correlations.

We constructed correlation heatmaps for sRAGE concentration across all study subjects, including COPD patients, exposed controls, and healthy controls. This comprehensive view highlights overall associations between sRAGE and variables such as age, BMI, lung function measures (FEV_1_, FVC, FEV_1_/FVC), symptom scores (mMRC, CAT), and inflammatory markers (NLR, PLR, CRP). It establishes the baseline relationships in the entire cohort, helping to understand broad trends of sRAGE in both diseased and non-diseased states (Figure 1A). The next correlation heatmap is restricted to the COPD group only. This focuses on worse lung function, higher symptom burden, and elevated inflammatory markers. Such patterns support the role of sRAGE as a biomarker indicating COPD severity and phenotype differentiation within affected individuals [Figure 1B]. The correlation heatmap for tobacco smoke- and biomass smoke-exposed controls (TS-control + BMS-control) without clinical COPD presents a subgroup analysis crucial for detecting early biomarker changes linked to environmental exposure before overt disease develops. It illuminates subtle associations between sRAGE and factors reflecting exposure dose and preclinical functional changes, potentially identifying subjects at high risk who may benefit from targeted preventive interventions [Figure 1C]. Finally, we present the correlation heatmap for healthy controls who lack significant smoke exposure or lung disease. This profile serves as a normative reference, showing physiological sRAGE interactions with demographic and clinical variables. The stable and higher sRAGE levels here contrast with the pathological patterns in the exposed and COPD groups, aiding in distinguishing disease-related biomarker alterations from normal variation [Figure 1D]. Detailed subgroup-wise correlation values between sRAGE and lung function parameters are provided in Supplementary Table S2.

**Figure 2:**
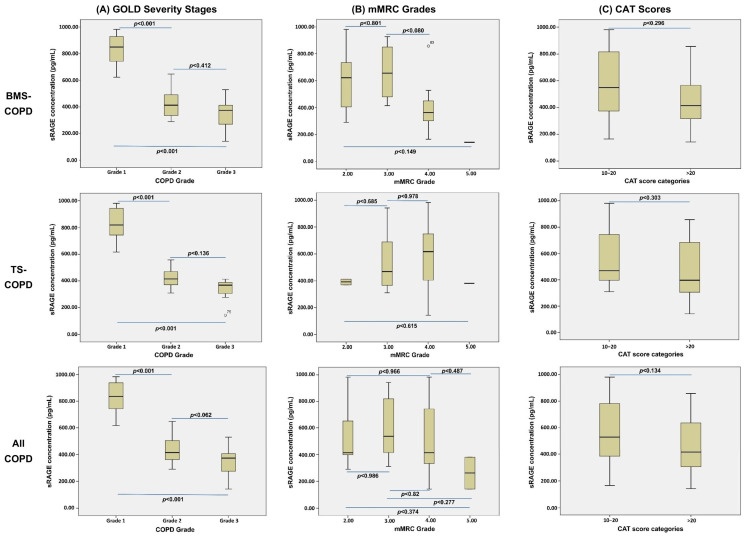
Comparative analysis of COPD severity and symptom burden in tobacco-smoke-related COPD (TS-COPD), biomass-smoke-related COPD (BMS-COPD), and the combined COPD cohort. The figure presents distribution and correlation patterns of serum soluble receptor for advanced glycation end-products (sRAGE) levels with (**A**) GOLD severity stages, (**B**) Modified Medical Research Council (mMRC) dyspnea grades, (**C**) COPD Assessment Test (CAT) scores. Abbreviations: GOLD: Global Initiative for Chronic Obstructive Lung Disease; mMRC: Modified Medical Research Council; CAT: COPD Assessment Test; sRAGE: Soluble Receptor for Advanced Glycation End-Products; COPD: Chronic Obstructive Pulmonary Disease; TS: Tobacco Smoke; BMS: Biomass Smoke.

The severity of COPD in the study groups was assessed using GOLD, mMRC, and CAT scoring systems. Both BMS-COPD (biomass-smoke-related COPD) and TS-COPD (tobacco-smoking-related COPD) patients demonstrated varying degrees of airflow limitation according to GOLD staging, as well as significant symptom burden reflected by higher mMRC and CAT scores. When considering all COPD patients together, these scores indicated moderate to severe disease and substantial symptoms impacting quality of life. Overall, both subgroups exhibited similar patterns of disease severity and symptomatic burden as captured by these standardized clinical measures (Figure 2).

**Figure 3:**
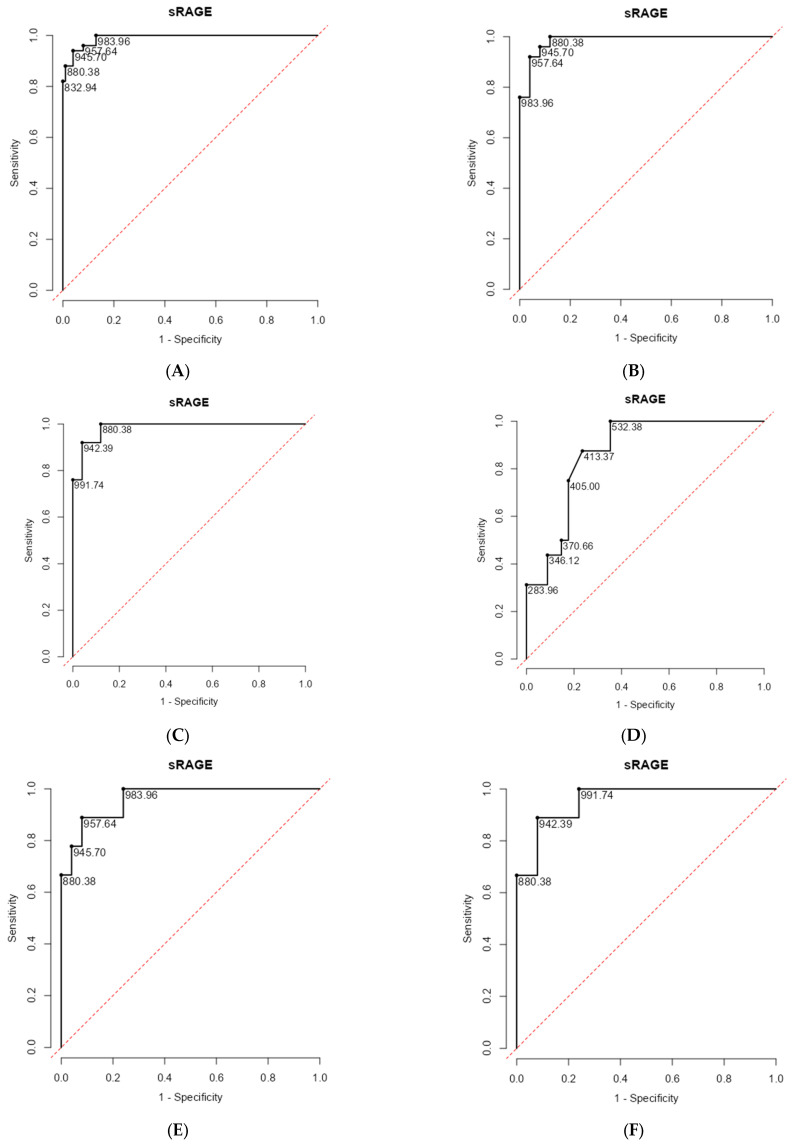
ROC curve for quantifying sRAGE levels in COPD and non-COPD participants (**A**), TS-COPD and TS-non-COPD participants (**B**), BMS-COPD and BMS-non-COPD participants (**C**), non-severe (mild + moderate) and severe COPD (**D**), TS-COPD (mild) and TS-non-COPD participants (**E**), BMS-COPD (mild) and BMS-non-COPD participants (**F**). Abbreviations: COPD: Chronic Obstructive Pulmonary Disease; TS: Tobacco Smoke; BMS: Biomass Smoke; sRAGE: Soluble Receptor for Advanced Glycation End Products.

Figure 3A shows that sRAGE discriminates COPD from non-COPD participants with an AUC of 0.990, sensitivity of 94%, and specificity of 96% at a 946 pg/mL cutoff, demonstrating diagnostic accuracy. Figure 3B highlights tobacco-smoke-related groups: sRAGE distinguishes TS-COPD from TS-non-COPD with an AUC of 0.986, a sensitivity of 92%, and a specificity of 96% at a 958 pg/mL cutoff. Figure 3C assesses biomass smoke exposure, where sRAGE achieves an AUC of 0.984, sensitivity of 100%, and specificity of 88% at an 880 pg/mL cutoff. Figure 3D compares non-severe versus severe COPD: here, sRAGE’s AUC is 0.866, with a sensitivity of 87.5% and a specificity of 76.5% at a 413 pg/mL cutoff, indicating moderate ability to classify disease severity. Figure 3E further evaluates mild COPD in the tobacco-exposed group: sRAGE distinguishes mild TS-COPD from TS-non-COPD with an AUC of 0.960, a sensitivity of 92%, and a specificity of 88.9% at a 957.6 pg/mL cutoff. Figure 3F presents mild COPD in biomass-exposed individuals, where sRAGE achieves an AUC of 0.956, with 92% sensitivity and 88.9% specificity at a 942.4 pg/mL cutoff, demonstrating strong diagnostic performance even in early disease across exposure types.

Serum sRAGE provides excellent diagnostic accuracy for COPD and its subtypes (AUCs > 0.98, high sensitivity and specificity), but shows only moderate accuracy (AUC 0.866) for differentiating between non-severe and severe COPD cases (Table 3).

Multivariable logistic regression showed that lower sRAGE levels were independently associated with COPD across most exposure comparisons, particularly in biomass-exposed groups (*p* < 0.05). Smoking pack-years remained the strongest predictor of COPD in tobacco-exposed subgroups (*p* < 0.001), whereas age significantly increased COPD risk in biomass-exposed groups only (*p* < 0.05). BMI was not a significant predictor in any model (Table 4).

## 4. Discussion

In this prospective, exposure-stratified cohort study, we demonstrate that serum soluble receptor for advanced glycation end-products (sRAGE) is significantly reduced in COPD, irrespective of exposure type, and is strongly associated with airflow obstruction, symptom burden, and disease severity. Our findings extend previous evidence from Western cohorts by confirming the diagnostic and severity-assessing potential of sRAGE in both tobacco- and biomass-related COPD phenotypes—an area of critical importance in LMICs, where non-smoking exposures remain under-recognized and under-investigated.

RAGE and its soluble isoform sRAGE play a central role in pulmonary inflammation, oxidative stress, and alveolar injury—hallmarks of COPD pathogenesis. Experimental and human studies suggest that ligand-induced activation of membrane-bound RAGE perpetuates chronic inflammation and matrix degradation in the lung, while sRAGE acts as a protective decoy receptor, buffering these signals. In the current study, both TS-COPD and BMS-COPD groups exhibited marked reductions in circulating sRAGE compared to exposure-matched and healthy controls, aligning with prior findings from the COPDGene and ECLIPSE cohorts that linked lower sRAGE to greater emphysematous destruction, rapid lung function decline, and increased mortality risk [8,16].

The inverse associations of sRAGE with CAT, mMRC, and SGRQ scores in our cohort reinforce its relevance not only as a pathophysiological marker but also as a surrogate for symptomatic and functional impairment. Notably, these correlations were robust across both tobacco- and biomass-related disease, suggesting that sRAGE may reflect a common final inflammatory pathway despite divergent exposure sources.

One of the most important observations of our study is the direct comparison of sRAGE in biomass-related versus tobacco-related COPD. Biomass exposure, primarily affecting women in LMICs, is known to induce a distinct airway-predominant COPD phenotype characterized by less emphysema and more small-airway inflammation and fibrosis, yet very few biomarker studies have included this population. Our finding that sRAGE is similarly reduced in biomass-exposed COPD patients suggests that the RAGE axis is active across phenotypes, even in the absence of classical smoking-related emphysematous change [17,18]. This finding expands the applicability of sRAGE beyond the emphysema-centric paradigm and underscores its potential utility in non-smoking populations.

In parallel, we observed elevated systemic inflammatory markers—particularly NLR—in BMS-COPD patients. This aligns with recent evidence suggesting that biomass exposure elicits more systemic and mucosal neutrophilic inflammation compared to tobacco smoke [19]. The modest inverse correlation between sRAGE and NLR in our study hints at a complex interplay between innate immune activation and sRAGE depletion, possibly through increased ligand burden or altered clearance mechanisms. While PLR did not reach significance, the trend reinforces the need to explore multi-marker inflammatory panels to complement sRAGE in future phenotyping algorithms.

ROC curve analyses revealed excellent discrimination between COPD and non-COPD groups (AUC = 0.990), with similarly high accuracy in both tobacco- and biomass-exposed subgroups. Notably, sRAGE also distinguished severe from non-severe COPD with good precision (AUC = 0.866), suggesting its potential use in severity stratification and referral prioritization. These results position sRAGE as a practical, accessible biomarker—especially relevant in primary care and LMIC settings, where spirometry is often unavailable or underutilized. The feasibility of serum-based ELISA assays, combined with the strong correlations with spirometric and symptomatic parameters, supports the integration of sRAGE into screening algorithms and community health workflows. Our findings support the inclusion of sRAGE in a new generation of COPD risk stratification tools, particularly in settings where tobacco is not the dominant exposure and traditional diagnostics are limited. Moreover, the dual inflammatory and structural signals captured by sRAGE may offer utility in identifying treatable traits and monitoring therapeutic response.

In addition, our multivariable logistic regression analysis demonstrated that lower sRAGE levels were independently associated with COPD across exposure subgroups, with a more pronounced effect in biomass-related COPD. Smoking pack-years remained a dominant predictor in tobacco-related COPD, whereas age contributed more significantly in biomass-exposed individuals. These findings further support the diagnostic value of sRAGE while highlighting exposure-specific influences on disease susceptibility and pathophysiology.

Although the present study provides strong evidence for the diagnostic relevance of sRAGE in COPD, its performance in differentiating COPD from other chronic respiratory diseases remains unknown. Asthma, interstitial lung disease (ILD), and bronchiectasis can also alter systemic inflammatory signaling, and therefore may influence sRAGE levels. The absence of disease control groups in our study limits the generalizability of the diagnostic cutoffs. Future work should include individuals with other respiratory conditions to determine the true differential diagnostic capability of sRAGE in real-world clinical practice.

While earlier studies such as SPIROMICS and COPDGene have consistently reported reduced sRAGE levels in association with emphysema severity and disease progression in tobacco-related COPD, our study extends this body of evidence in important and novel ways [16,17]. Most notably, it is among the first to demonstrate that sRAGE suppression occurs to a similar extent in biomass-smoke-related COPD—a phenotype that remains underrepresented in global biomarker research despite its high prevalence in low- and middle-income countries [18,19]. By including exposure-matched control groups for both tobacco and biomass smoke, our design enables a clearer distinction between exposure effects and disease-specific changes in sRAGE levels. Moreover, by correlating sRAGE with validated clinical tools such as the CAT score, mMRC dyspnea scale, and SGRQ, we establish the biomarker’s relevance not only to physiological impairment but also to patient-reported symptom burden and health status. This reinforces the potential clinical utility of sRAGE as a multidimensional disease indicator.

COPD is increasingly recognized as a multisystem disorder, in which local pulmonary injury triggers systemic responses via oxidative stress, inflammation, endothelial dysfunction, and metabolic dysregulation. The receptor for advanced glycation end-products (RAGE) axis lies at the heart of this cross-organ communication. RAGE is a pattern-recognition receptor expressed on alveolar epithelial cells, vascular endothelium, and immune cells, which binds diverse ligands—advanced glycation end-products (AGEs), HMGB1, S100/calgranulins—triggering NF-κB-driven inflammatory cascades and reactive oxygen species generation [20]. In healthy physiology, soluble RAGE (sRAGE) acts as a decoy receptor, neutralizing these ligands before they engage a membrane-bound receptor. Reduced levels of sRAGE in COPD therefore may reflect a diminished homeostatic buffer against pro-inflammatory stimuli, contributing to systemic vascular injury, endothelial activation, and propagation of comorbid cardiovascular disease [21].

Beyond the pulmonary compartment, accumulating evidence indicates that dysregulation of the RAGE–sRAGE axis contributes to a spectrum of systemic consequences in COPD. The chronic activation of RAGE signaling amplifies oxidative and inflammatory stress in vascular endothelium, leading to endothelial dysfunction, arterial stiffness, and atherosclerotic progression—mechanisms strongly implicated in the elevated cardiovascular morbidity observed in COPD patients [22,23,24]. In parallel, other circulating mediators including GDF-15, IL-6, and skeletal-muscle-derived myokines have been identified as systemic biomarkers that reflect inflammation-, metabolism-, and aging-related pathways contributing to COPD’s extrapulmonary manifestations [25]. Integrating sRAGE within this broader biomarker network suggests that sRAGE may participate in a shared molecular response to systemic immune–metabolic stress.

sRAGE deficiency, by reducing the extracellular neutralization of pro-inflammatory ligands, may permit their spillover into the systemic circulation, promoting low-grade inflammation that extends to skeletal muscle, adipose tissue, and the liver. This contributes to peripheral muscle wasting, insulin resistance, and metabolic syndrome, all of which are recognized extrapulmonary manifestations of COPD [7,26]. RAGE-mediated oxidative injury has also been implicated in renal microvascular remodeling and endothelial permeability, suggesting that reduced sRAGE may reflect a shared molecular pathway linking COPD to multi-organ dysfunction [27].

Together, these findings position sRAGE not only as a biomarker of pulmonary injury but as a sentinel indicator of systemic inflammatory and metabolic derangement that bridges the lung with cardiovascular and metabolic comorbidities characteristic of advanced COPD.

Finally, we provide population-specific diagnostic cutoffs with strong sensitivity and specificity for both tobacco- and biomass-related COPD, offering practical thresholds that can be translated into clinical or community-level screening protocols in resource-constrained settings [18,19]. Together, these contributions position sRAGE as a promising and adaptable biomarker across diverse COPD phenotypes and exposure contexts.

### Strengths and Limitations

This study has several notable strengths. First, it is among the few to include both tobacco- and biomass-smoke-related COPD within a unified biomarker framework, addressing a critical knowledge gap in the global COPD literature [28]. The inclusion of well-characterized, exposure-matched control groups alongside healthy, unexposed controls allows for a more precise evaluation of disease-specific alterations in sRAGE levels, independent of background exposure. Second, the prospective design and standardized phenotyping—encompassing spirometry, symptom scores, inflammatory indices, and serum biomarker assessment—facilitated comprehensive cross-domain comparisons. The use of validated symptom instruments (CAT, mMRC, and SGRQ) adds clinical relevance to the biomarker associations, while the ROC analyses provide actionable diagnostic cutoffs with strong discriminatory power [29]. Importantly, the study was conducted in a real-world, resource-limited setting, enhancing its applicability to primary care and community health programs in low- and middle-income countries.

Nonetheless, several limitations should be acknowledged. Although major systemic comorbidities were excluded to reduce confounding, this may limit generalizability to real-world COPD populations where such comorbidities are highly prevalent. Moreover, disease control groups such as asthma or ILD were not included. As a result, the diagnostic performance reported here reflects discrimination between COPD and healthy or exposure-matched controls, and the findings should be interpreted as exploratory. The single-center design may limit generalizability to other geographic or healthcare contexts, particularly regions with differing patterns of biomass fuel use or comorbidities. Although the sample size was sufficient for group-level comparisons and ROC analysis, subgroup analyses (e.g., within GOLD stages) may have been underpowered. The cross-sectional nature of the study precludes causal inference or assessment of temporal changes in sRAGE in response to disease progression or treatment. Additionally, potential confounding factors—such as diet, environmental co-exposures, or metabolic status—that may influence sRAGE levels were not comprehensively assessed [12]. Despite these limitations, this study provides foundational evidence for the utility of sRAGE as a diagnostic and severity biomarker across diverse COPD phenotypes and lays the groundwork for larger, multicenter, and longitudinal studies.

A previous study provides compelling evidence that serum sRAGE is a robust, non-invasive biomarker for COPD diagnosis and severity assessment, showing reduced sRAGE levels in affected individuals [30]. Circulating sRAGE levels have correlated with emphysema severity and complications such as chronic cor pulmonale in smokers, underscoring their pathophysiological relevance in tobacco-related COPD [21]. MacNee described the underlying pathology and inflammatory processes in COPD, supporting the biological plausibility of sRAGE as a marker reflecting disease mechanisms [31]. Together, these studies align with our findings of consistent sRAGE suppression across tobacco- and biomass-exposed COPD patients, and highlight its high diagnostic accuracy and potential utility in resource-limited settings, warranting larger longitudinal studies to validate sRAGE as part of a multi-biomarker approach for early detection, risk stratification, and personalized COPD management.

## 5. Conclusions

This study demonstrates that serum sRAGE is significantly reduced in COPD patients regardless of tobacco or biomass smoke exposure, highlighting its role as a versatile biomarker across distinct COPD phenotypes. Strong correlations of sRAGE with lung function decline, symptom severity, and systemic inflammation underscore its potential for early diagnosis and disease severity assessment. The excellent diagnostic accuracy of sRAGE supports its practical application, especially in low-resource settings where spirometry access is limited. These findings lay the groundwork for integrating sRAGE into COPD screening and management strategies, with further large-scale and longitudinal studies warranted to validate its clinical utility.

## Figures and Tables

**Table 1:** Demography and clinical characteristics of the study cohort.

Variable	TS-COPD (*n* = 25)	TS-CONTROL (*n* = 25)	BMS-COPD (*n* = 25)	BMS-Control (*n* = 25)	Healthy Controls (*n* = 50)
Age (years) (Mean ± SD)	62.00 ± 6.50	56.64 ± 9.13	68.00 ± 7.74	61.16 ± 10.26	50.52 ± 9.92
BMI (Mean ± SD)	21.60 ± 4.13	22.39 ± 3.36	22.31 ± 2.98	23.56 ± 2.62	23.62 ± 2.33
Gender					
Male (*n*)	25	25	-	-	25
Female (*n*)	-	-	25	25	25
FEV_1_/FVC Ratio (Mean ± SD)	0.59 ± 0.11	0.79 ± 0.05	0.60 ± 0.08	0.80 ± 0.04	0.81 ± 0.05
FVC % Predicted (Mean ± SD)	83.56 ± 20.33	100.16 ± 10.98	78.64 ± 21.77	102.50 ± 12.61	100.90 ± 16.27
FEV_1_% Predicted (Mean ± SD)	62.32 ± 21.42	97.12 ± 12.53	62.40 ± 20.15	101.80 ± 13.37	99.56 ± 14.99
Biomass Exposure Years (Median, IQR)	–	–	23 (17–28)	15 (10–16)	–
BMEI (Median, IQR)	–	–	90 (75–102)	48 (40–60)	–
Pack-Years (Median, IQR)	41 (26–55)	23 (13–41)	–	–	–
Smoking Index (Median, IQR)	810 (525–1100)	450 (256–816)	–	–	–
GOLD Severity Grading (*n*)					
I	9		9		
II	9		7		
III	7		9		
CAT Score (Median, IQR)	20 (16–23)	7 (4–10)	17 (16–21)	8 (4–9)	0 (0–0)
mMRC Grade (Median, IQR)	3 (3–4)	1 (1–1)	3 (2–4)	1 (1–1)	0 (0–0)
SGQRC Symptoms (Median, IQR)	56.80 (46.68–71.72)	0 (0–25.36)	53.57 (44.93–72.04)	0 (0–10.95)	0 (0–0)
SGQRC Activity (Median, IQR)	67.87 (44.90–77.00)	0 (0–7.74)	67.87 (54.01–84.21)	0 (0–0)	0 (0–0)
SGQRC Impact (Median, IQR)	19.30 (14.33–31.96)	2.54 (2.54–5.09)	29.99 (19.30–48.00)	2.54 (2.54–5.09)	0 (0–0)
SGQRC Total (Median, IQR)	44.58 (31.02–51.09)	3.25 (1.31–10.69)	45.78 (41.41–57.14)	3.25 (1.31–6.98)	0 (0–0)
NLR (Mean ± SD)	2.56 ± 2.28	1.85 ± 1.06	7.92 ± 11.33	1.76 ± 1.10	1.87 ± 0.94
PLR (Mean ± SD)	115.88 ± 44.45	129.99 ± 66.40	192.50 ± 145.09	115.22 ± 54.57	128.06 ± 43.13
sRAGE (Mean ± SD)	545 ± 238	1207 ± 228	540 ± 251	1237 ± 266	1462 ± 347

Abbreviations: BMI: Body Mass Index; FEV_1_: Forced Expiratory Volume In One Second; FVC: Forced Vital Capacity; COPD: Chronic Obstructive Pulmonary Disease; TS: Tobacco Smoke; BMS: Biomass Smoke; BMEI: Biomass Exposure Index; GOLD: Global Initiative For Chronic Obstructive Lung Disease; CAT: COPD Assessment Test; mMRC: Modified Medical Research Council; SGQRC: COPD-Specific Version of the St. George’s Respiratory Questionnaire; IQR: Interquartile Range (25th percentile; 75th percentile); NLR: Neutrophil to Lymphocyte Ratio; PLR: Platelet to Lymphocyte Ratio; SD: Standard Deviation of Mean; sRAGE: Soluble Receptor for Advanced Glycation End-Products.

**Table 2:** Correlation analysis of sRAGE with clinical and pulmonary function parameters.

Parameter	sRAGE Concentration	
	Pearson Correlation	*p*-Value
FEV_1_/FVC Ratio	0.748	<0.001
FVC Percent Predicted	0.543	<0.001
FEV_1_ Percent Predicted	0.694	<0.001
CAT Score	−0.768	<0.001
mMRC Grade	−0.791	<0.001
SGQRC Total	−0.803	<0.001
NLR	−0.265	<0.001
PLR	−0.145	0.072

Abbreviations: FEV_1_: Forced Expiratory Volume in One Second; FVC: Forced Vital Capacity; CAT: COPD Assessment Test; mMRC: Modified Medical Research Council; SGQRC: COPD-Specific Version of the St. George’s Respiratory Questionnaire; NLR: Neutrophil to Lymphocyte Ratio; PLR: Platelet to Lymphocyte Ratio; sRAGE: Soluble Receptor for Advanced Glycation End-Products.

**Table 3:** Diagnostic performance of sRAGE in COPD patient subgroups based on ROC curve analysis.

	sRAGE Cutoff Point	Sensitivity (%)	Specificity (%)	PPV (%)	NPV (%)	Youden’s Index	AUC
Patients with COPD and without COPD	946	0.940	0.960	0.922	0.970	1.90	0.990
Patients with TS COPD and TS without COPD	958	0.920	0.960	0.958	0.923	1.88	0.986
Patients with BMS COPD and BMS without COPD	880	1.000	0.880	0.893	1.000	1.88	0.984
Patients with non-severe (mild and moderate) and severe COPD	413	0.875	0.7647	0.636	0.929	1.64	0.866
Patients with TS COPD (mild) and TS without COPD	957.6	0.920	0.889	0.479	0.990	0.809	0.960
Patients with BMS COPD (mild) and BMS without COPD	942.4	0.920	0.889	0.479	0.990	0.809	0.956

Abbreviations: sRAGE: Soluble Receptor for Advanced Glycation End-Products; COPD: Chronic Obstructive Pulmonary Disease; ROC: Receiver Operating Characteristic; TS: Tobacco Smoke; BMS: Biomass Smoke; PPV: Positive Predicative Value; NPV: Negative Predicative Value; AUC: Area Under the Curve.

**Table 4:** Multivariable logistic regression analysis of sRAGE and clinical covariates for COPD diagnosis across exposure subgroups.

			95% CI				
COPD Status	Predictor	Estimate	Lower	Upper	SE	Z	*p*
Patients with TS COPD and without COPD	Age in yrs	0.084	−0.421	0.589	0.258	0.325	0.745
	BMI (kg/m^2^)	−0.376	−2.193	1.442	0.927	−0.405	0.685
	sRAGE (pg/mL)	−0.023	−0.046	0.000	0.012	−1.958	0.050
	Smoking pack-years	−1.996	−2.407	−1.585	0.210	−9.521	<0.001
Patients with TS without COPD-NORMAL	Age in yrs	0.078	−0.433	0.588	0.260	0.298	0.766
	BMI (kg/m^2^)	−0.314	−2.179	1.552	0.952	−0.329	0.742
	sRAGE (pg/mL)	−0.004	−0.027	0.020	0.012	−0.298	0.765
	Smoking pack-years	−2.032	−2.458	−1.606	0.217	−9.358	<0.001
Patients with BMS COPD and without COPD	Age in yrs	0.244	0.033	0.456	0.108	2.265	0.024
	BMI (kg/m^2^)	0.151	−0.352	0.655	0.257	0.589	0.556
	sRAGE (pg/mL)	−0.029	−0.051	−0.008	0.011	−2.647	0.008
	Smoking pack-years	1.009	0.797	1.221	0.108	9.313	<0.001
Patients with BMS without COPD-NORMAL	Age in yrs	0.107	0.045	0.169	0.032	3.361	<0.001
	BMI (kg/m^2^)	0.009	−0.219	0.238	0.116	0.082	0.935
	sRAGE (pg/mL)	−0.002	−0.004	−0.000	0.001	−2.314	0.021
	Smoking pack-years	0.424	0.360	0.489	0.033	12.916	<0.001
Patients with TS COPD and TS without COPD	Age in yrs	0.010	−0.117	0.137	0.065	0.152	0.879
	BMI (kg/m^2^)	−0.041	−0.599	0.517	0.285	−0.143	0.886
	sRAGE (pg/mL)	−0.021	−0.033	−0.008	0.006	−3.212	0.001
	Smoking pack-years	0.035	−0.044	0.115	0.041	0.868	0.385
Patients with BMS COPD and BMS without COPD	Age in yrs	0.119	−0.069	0.307	0.096	1.239	0.215
	BMI (kg/m^2^)	0.078	−0.386	0.541	0.237	0.329	0.742
	sRAGE (pg/mL)	−0.025	−0.044	−0.006	0.010	−2.529	0.011
	Smoking pack-years	1.424	1.228	1.621	0.100	14.194	<0.001

Abbreviations: sRAGE: Soluble Receptor for Advanced Glycation End-Products; COPD: Chronic Obstructive Pulmonary Disease; TS: Tobacco Smoke; BMS: Biomass Smoke; BMI: Body Mass Index; kg/m^2^: Kilograms per square meter; pg/mL: Picograms per milliliter.

## Data Availability

The data presented in this study are available on request from the corresponding author. The data are not publicly available due to privacy or ethical restrictions.

## Supplementary Materials

The following supporting information can be downloaded at: https://www.mdpi.com/article/10.3390/diagnostics15222910/s1, Table S1: Gender-based stratification analysis of serum soluble receptor for advanced glycation end-products (sRAGE) concentrations [median, interquartile range (IQR)] in different subgroups of study subjects, along with corresponding statistical analysis [non-parametric Kruskal–Wallis test followed by Mann–Whitney *U* test (two-tailed), when appropriate; *p* < 0.05 was considered significant]; Table S2: Correlation of serum concentrations of sRAGE with post-bronchodilator (post-BD) tests of forced expiratory volume in one second (FEV_1_% predicted), forced vital capacity (FVC % predicted), and FEV_1_/FVC among the different subgroups of study subjects. Two-tailed non-parametric Pearson test used to analyze the correlation. *p* < 0.05 was considered statistically significant.

## Abbreviations

The following abbreviations are used in this manuscript:

Abbreviation	Full Form
ANOVA	Analysis of Variance
ATS/ERS	American Thoracic Society/European Respiratory Society
AUC	Area Under the Curve
BMEI	Biomass Exposure Index
BMS	Biomass Smoke
CAT	COPD Assessment Test
COPD	Chronic Obstructive Pulmonary Disease
FEV_1_	Forced Expiratory Volume in One Second
FVC	Forced Vital Capacity
GOLD	Global Initiative for Chronic Obstructive Lung Disease
IQR	Interquartile Range (25th percentile, 75th percentile)
LMICs	Low- and Middle-Income Countries
mMRC	Modified Medical Research Council
NLR	Neutrophil-to-Lymphocyte Ratio
NPV	Negative Predictive Value
PFT	Pulmonary Function Test
PLR	Platelet-to-Lymphocyte Ratio
PPV	Positive Predictive Value
SD	Standard Deviation
SGQRC	COPD-Specific Version of the St. George’s Respiratory Questionnaire
sRAGE	Soluble Receptor for Advanced Glycation End-Products
TS	Tobacco Smoke
