# Supplementary material for: Reduced Serum sRAGE Levels Identify COPD and Reflect Disease Severity: Findings from a Cross-Sectional Study in India
Source: Diagnostics (Basel). 2025 Nov 17;15(22):2910. doi: 10.3390/diagnostics15222910 (PMC12651461; doi:10.3390/diagnostics15222910)
Supplement: Supplementary file 1 [file diagnostics-15-02910-s001.zip › diagnostics-3950446-supplementary.pdf]

diagnostics

MDPI

Table S1. Gender based stratification analysis of serum soluble Receptor for Advanced Glycation End-products (sRAGE) concentrations [median, interquartile range (IQR)] in Different sub-groups of the study subjects along with corresponding statistical analysis [non-parametric Kruskal-Wallis followed by Mann Whitney U test (two tailed), when appropriate; p<0.05 was considered as significant.

**A. All study subjects**					
**Study groups**	**TS-COPD**	**TS-CONTROL**	**BS-COPD**	**BS-Control**	**Healthy Control**
Total (n)	25	25	25	25	50
Male	25	25	0	0	25
Female	0	0	25	25	25
sRAGE (ng/mL) (Median, IQR)	463 (372–743)	1,190 (986–1,328)	480 (363–743)	1,206 (1,002–1,388)	1,354 (1,203–1,805/1,648) *
Kruskal-Wallis test	<.001				
Mann-Whitney test (p)	Pairwise Comparison			w	p
	TS-COPD vs TS-CONTROL			-8.328	<.001
	TS-COPD vs BS-CONTROL			-8.219	<.001
	TS-COPD vs HEALTHY CONTROLS			-9.872	<.001
	BS-COPD vs BS-CONTROL			-8.301	<.001
	BS-COPD vs TS-CONTROL			8.356	<.001
	BS-COPD vs HEALTHY CONTROLS			9.856	<.001
	TS-COPD vs BS-COPD			0.165	1.000
	BS-CONTROL vs TS-CONTROL			-0.192	1.000
	HEALTHY CONTROLS vs TS-CONTROL			-4.451	0.014
	BS-CONTROL vs HEALTHY CONTROLS			3.863	0.050

**B. Gender stratification of COPD and healthy control**				
Study groups	**COPD**		**Healthy Control**	
	Male	Female	Male	Female
Total (n)	25	25	25	25
sRAGE (ng/m) (Median, IQR)	463 (372–743)	480 (363–743)	1,356 (1,235–1,805)	1,351 (1,203–1,648)

Mann-Whitney test	Copd vs Healthy controls	<.001		
**C. GOLD stage (all COPD subjects)**				
COPD Grade	I	II	III	
Total (n)	18	16	16	
Female (n)	9	7	9	
Male (n)	9	9	7	
sRAGE (ng/mL) Median (IQR)	835 (743–934)	413 (362–486)	371 (276–401)	
Kruskal-Wallis test	<.001			
Mann-Whitney test (*p*)	Pairwise Comparison		W	*p*
	I vs II		-6.93	<.001
	I vs III		-7.03	<.001
	II vs III		-2.94	0.095
**D. GOLD stage (TS COPD)**				
COPD Grade	I	II		III
Total (n)	9	9		7
sRAGE (ng/mL) Median (IQR)	819 (743–943)	414 (372–469)		369 (305–389)
Kruskal-Wallis test	<.001			
Mann-Whitney test (p)	Pairwise Comparison	W		*p*
	I vs II	-5.06		0.001
	I vs III	-4.72		0.002
	II vs III	-2.85		0.109
**E. GOLD stage (BS COPD)**				
COPD Grade	I	II		III
Total (n)	9	7		9
sRAGE (ng/mL) Median (IQR)	850 (743–928)	413 (337–492)		373 (270–413)
Kruskal-Wallis test	<.001			

Mann-Whitney test (p)	Pairwise Comparison	W	*p*
	I vs II	-4.57	0.004
	I vs III	-5.06	0.001
	II vs III	-1.50	0.540
**F. CAT Score (all COPD subjects)**			
**CAT Score**	**<10 (Low)**	**10–20 (Medium)**	**>20 (High)**
Total (*n* = 50)	—	35	15
Male (*n* =25)	—	17	8
Female (*n* = 25)	—	18	7
sRAGE (median, IQR)	—	528 (385–780)	414 (305–634)
Kruskal-Wallis test	0.162		
Mann-Whitney test (*p*)	Pairwise Comparison	**W**	***p***
	**10-20 vs >20**	-1.98	0.162
**G. CAT Score (TS-COPD subjects)**			
**CAT Score**	**<10 (Low)**	**10–20 (Medium)**	**>20 (High)**
Total (*n* = 25)	—	17	8
sRAGE (median, IQR)	—	469 (398–743)	397 (319–650)
Kruskal-Wallis test	0.294		
Mann-Whitney test (*p*)	Pairwise Comparison	**W**	***p***
	**10-20 vs >20**	-1.48	0.294
**H. CAT Score (BS-COPD subjects)**			
**CAT Score**	**<10 (Low)**	**10–20 (Medium)**	**>20 (High)**
Total (*n* = 25)	—	18	7
sRAGE (median, IQR)	—	549 (379–798)	414 (316–566)
Kruskal-Wallis test	0.364		
Mann-Whitney test (*p*)	Pairwise Comparison	**W**	***p***
	**10-20 vs >20**	-1.28	0.364
I. **SGRQ-C (total) (all COPD subjects)**			
**SGRQ-C (total)**	**<10 (Low)**	**10–20 (Medium)**	**>20 (High)**
Total (*n* = 50)	—	02	48
Male (*n* =25)	—	2	23
Female (*n* = 25)	—	0	25
sRAGE (median, IQR)	—	786 (769–802)	438 (368–738)
Kruskal-Wallis test	0.151		
Mann-Whitney test (*p*)		**W**	***p***
	**10-20 vs >20**	-2.03	0.151
J. **SGRQ-C (total) (TS-COPD)**			
**SGRQ-C (total)**	**<10 (Low)**	**10–20 (Medium)**	**>20 (High)**
Total (*n* = 25)	—	2	23
sRAGE (median, IQR)	—	786 (769–802)	414 (371–676)

Kruskal-Wallis test	0.133			
Mann-Whitney test (*p*)		**W**		***p***
	**10-20 vs >20**	-2.13		0.133
K. **SGRQ-C (total) (BS-COPD)**				
**SGRQ-C (total)**	**<10 (Low)**	**10–20 (Medium)**		**>20 (High)**
Total (*n* = 25)	—	0		25
sRAGE (median, IQR)	—	0		480 (363–743)
Kruskal-Wallis test	—			
Mann-Whitney test (*p*)		**W**		***p***
	**10-20 vs >20**	—		—
**L. mMRC GRADE (all COPD subjects)**				
**mMRC GRADE**	1	**2**	**3**	**4**
Total (*n* = 50)	—	13	17	20
Male (*n* =25)	—	2	11	12
Female (*n* = 25)	—	11	6	8
sRAGE (median, IQR)	—	413 (398–653)	537 (414–819)	405 (319–738)
Kruskal-Wallis test	0.373			
Mann-Whitney test (*p*)	Pairwise Comparison	**W**		***p***
	Grade 2 vs Grade 3	0.770		0.849
	Grade 2 vs Grade 4	-1.252		0.650
	Grade 3 vs Grade 4	-1.854		0.389
**M. mMRC GRADE (TS-COPD subjects)**				
**mMRC GRADE**	1	**2**	**3**	**4**
Total (*n* = 25)	—	2	11	12
sRAGE (median, IQR)	—	391 (380–402)	469 (366–688)	515 (393–746)
Kruskal-Wallis test	0.615			
Mann-Whitney test (*p*)	Pairwise Comparison	**W**		***p***
	Grade 2 vs Grade 3	1.117		0.710
	Grade 2 vs Grade 4	1.422		0.574
	Grade 3 vs Grade 4	0.348		0.967
**N. mMRC GRADE (BS-COPD subjects)**				
**mMRC GRADE**	1	**2**	**3**	**4**
Total (*n* = 25)	—	11	6	8
sRAGE (median, IQR)	—	622 (405–734)	656 (502–823)	348 (243–412)
Kruskal-Wallis test	0.044			
Mann-Whitney test (*p*)	Pairwise Comparison	**W**		***p***
	Grade 2 vs Grade 3	1.14		0.700
	Grade 2 vs Grade 4	-2.80		0.117
	Grade 3 vs Grade 4	-3.10		0.072

Table S2. Correlation of serum concentrations of sRAGE with post- bronchodilator (post-BD) test of forced expiratory volume in one second (FEV1% predicted), forced vital capacity (FVC % predicted), and FEV1/FVC among the different sub-groups of the study subjects Two tailed non-parametric Pearsons test was used to analyze the correlation. *p*<0.05 was considered as statistically significant.

Category	N	FEV_1 (post-BD)	FVC (post-BD)	FEV_1/FVC (Post-BD)
**ALL SUBJECTS**	150	r = 0.663<br>*p* = <.001	r = 0.483<br>*p* = <.001	r = 0.735<br>*p* = <.001
**COPD (all)**	50	r = 0.795<br>*p* < 0.001	r = 0.766<br>*p* < 0.001	r = 0.509<br>*p* < 0.001
**BMS-COPD**	25	r = 0.738<br>*p* < 0.001	r = 0.820<br>*p* < 0.001	r = 0.345<br>ns *p* = 0.092
**TS-COPD**	25	r = 0.853<br>*p* < 0.001	r = 0.713<br>*p* < 0.001	r = 0.654<br>*p* < 0.001
**NON-COPD (TS CONTROL+ BS CONTROL+ HEALTHY CONTROLS)**	100	r = -0.096<br>*p* = 0.341	r = -0.136<br>*p* = 0.179	r = 0.117<br>*p* = 0.247
**(TS CONTROL+ BS CONTROL)**	50	r = 0.124<br>*p* = 0.392	r = 0.034<br>*p* = 0.817	r = 0.150<br>*p* = 0.298
**HEALTHY CONTROLS**	50	r = -0.247<br>*p* = 0.084	r = -0.228<br>*p* = 0.111	r = 0.010<br>*p* = 0.946
**MALE HEALTHY CONTROLS**	25	r = -0.091<br>*p* = 0.665	r = -0.055<br>*p* = 0.793	r = -0.041<br>*p* = 0.847
**FEMALE HEALTHY CONTROLS**	25	r = -0.391<br>*p* = 0.053	r = -0.398<br>*p* = 0.049	r = 0.074<br>*p* = 0.727
